# Exploring ocular fundus morphology in relation to growth in adolescents born moderate‐to‐late preterm

**DOI:** 10.1111/aos.70011

**Published:** 2025-10-03

**Authors:** Alexandra Lind, Sara Ali, Titus Ovik, Zoran Popovic, Eva Aring, Jovanna Dahlgren, Marita Andersson Grönlund

**Affiliations:** ^1^ Department of Clinical Neuroscience Institute of Neuroscience and Physiology, Sahlgrenska Academy at the University of Gothenburg Gothenburg Sweden; ^2^ Department of Paediatrics Region Västra Götaland, Sahlgrenska University Hospital Gothenburg Sweden; ^3^ Department of Ophthalmology Region Västra Götaland, Sahlgrenska University Hospital Mölndal Sweden; ^4^ Department of Paediatrics Institute of Clinical Sciences, Sahlgrenska Academy, University of Gothenburg Gothenburg Sweden; ^5^ Department of Ophthalmology, Faculty of Medicine and Health Örebro University Örebro Sweden

**Keywords:** Adolescence, insulin‐like growth factor‐I, macular retinal thickness, moderate‐to‐late preterm, optical coherence tomography and retinal nerve fibre layer

## Abstract

**Purpose:**

To study ocular fundus morphology and its relation to growth in adolescents born moderate‐to‐late preterm (MLP) and full term.

**Methods:**

This prospective and population‐based cohort study included 50 MLP adolescents (26 girls, mean age 16.5 years) and 50 full‐term controls (30 girls, mean age 16.7 years). Optical coherence tomography measurements were studied in relation to gestational age, auxological data, insulin‐like growth factor‐I (IGF‐I) and IGF‐binding protein 3 (IGFBP‐3).

**Results:**

The MLP group showed an increased central macular retinal thickness (MRT) compared with controls in right eye (RE) (249.7 ± 21.0 vs. 239.9 ± 16.4 μm, *p* = 0.019). Moreover, the MLP group showed a thinner total peripapillary retinal nerve fibre layer (ppRNFL) thickness in RE (104.3 ± 8.5 vs. 109.1 ± 8.3, *p* = 0.0011). Nasal ppRNFL thickness was thinner in both RE (79.4 ± 13.2 vs. 85.0 ± 10.8, *p* = 0.0012) and left eye (LE) (77.0 ± 13.8 vs. 81.7 ± 13.4, *p* = 0.025) compared with controls. A weak association between total ppRNFL thickness and IGF‐I levels was found (RE, *r* = 0.28, *p* = 0.032; LE, *r* = 0.27, *p* = 0.048), as well as between central MRT and a ratio between IGF‐I and IGFBP‐3 levels (RE, *r* = 0.30, *p* = 0.022). Additionally, there was a correlation between optic cup/disc area ratio and birth weight (RE: *r* = −0.44, *p* = 0.0006; LE: *r* = −0.30, *p* = 0.026).

**Conclusion:**

The present study suggests that growth and MLP birth may impact ocular fundus morphology. The MLP adolescents were shown to have thinner ppRNFL thickness and greater MRT, compared with full‐term controls. Furthermore, a weak association between these structures and growth factors was found. In addition, the current study proposes that birth weight may impact optic disc parameters.

## 1 INTRODUCTION

As early as 22 days after gestation, the eye begins to form as an optic sulcus in the forebrain. By day 24, the optic stalk is evident together with lateral evagination of the neural tube, the optic vesicle. Approximately five days later, the distal part of the optic vesicle transforms into the optic cup. The inner wall of the optic cup develops into the neural retina, whereas the outer wall develops into the retinal pigmented epithelium. As axons from the neural retina pass through the optic stalk, the optic nerve begins to form (Schoenwolf et al., 2015).

The retina including all cell layers is evident in the 8th gestational month. The foveal development begins at gestational week 22, with pit formation of the fovea occurring by gestational week 24. This process involves the migration of inner retinal layers to the periphery and outer retinal layers to the centre (Hendrickson & Yuodelis, 1984). After week 28, a distinct foveal pit is present and cone photoreceptors begin to elongate. After birth, the pit becomes wider and deeper, cone packing density increases and the foveal outer nuclear layer thickens (Hendricson et al., 2012). A few months after birth, the avascular fovea only consists of cone photoreceptors (Schoenwolf et al., 2015).

Preterm birth may halt macular development, leading to a migration disturbance of foveal structures. Consequently, individuals born preterm exhibit migration disturbances impacting retinal layers in the macula, such as reduced foveal depth and persistence of inner retinal layers (Fieß et al., 2022; Hendrickson & Yuodelis, 1984; Park & Oh, 2012). This phenomenon, among other ophthalmological complications, is well known in children born extremely (gestational age (GA) <28 weeks) and very (GA < 32 weeks) preterm, with or without the presence of retinopathy of prematurity (ROP) (Åkerblom et al., 2011; Hellström et al., 2000; Holmström et al., 2014).

Less is known of the ophthalmological development in the largest group of preterm children, namely the children born moderate‐to‐late preterm (MLP). Moderate preterm birth is defined as GA 32–33 weeks, whereas late preterm birth is defined as GA 34–36 weeks (Ohuma et al., 2023). Interest in studying the risks associated with being born MLP has surged in the last decade. Being born MLP significantly impacts overall health, with MLP infants facing increased susceptibility to respiratory dysfunction and infections, among other complications, despite advances in perinatal care (Altman et al., 2011). Later in life, they also have an increased risk of neurodevelopmental disabilities, psychiatric disorders and behavioural problems (Johnson et al., 2015; Lindström et al., 2009; Van Baar et al., 2009). Furthermore, prematurity, in children born MLP and others, has been linked to increased cardiovascular disease risk, including elevated blood pressure (Kerkhof et al., 2012).

Previous studies from our research group have shown that MLP individuals, with no history of ROP, exhibit a higher prevalence of ophthalmological findings at birth and throughout 5, 8, 10, 12 and 16 years of age, compared with full‐term controls. The ophthalmological findings include abnormal retinal vascularization, impaired eye motility, heterophoria, refractive errors, reduced visual evoked potential amplitudes and smaller foveal avascular zone (FAZ) area (Allvin et al., 2014; Lind et al., 2018; Ovik et al., 2024; Raffa et al., 2015, 2016, 2017).

The association between ocular development and general growth has been previously studied. Insulin‐like growth factor‐I (IGF‐I) is essential for foetal development, especially in the third trimester, supporting central nervous system development, including neurogenesis, myelination, differentiation, synaptogenesis and angiogenesis (Hwa et al., 2013). Levels of IGF‐I play an important role in central nervous system development (O'Kusky & Ping, 2012), where increased IGF‐I levels have been associated with increased brain volume in infants born extremely preterm (Hellström et al., 2022). The main circulating binding protein, insulin‐like growth factor‐binding protein 3 (IGFBP‐3), enhances the half‐life of IGF‐I (Nguyen et al., 2013). Receptors of IGF‐I have been discovered in retinal microvascular cells, emphasizing the potential significance of IGF‐I in ocular development (Nguyen et al., 2013). Moreover, IGF‐I has been linked to retinal vascularization (Allvin et al., 2014; Hellström et al., 2003; Lind et al., 2018) and choroidal thickness (Zhang et al., 2018). In addition, birth growth parameters, such as weight and head circumference (HCF), have been shown to be associated with ophthalmological changes, including the optic disc, retinal nerve fibre layer (RNFL), macular retinal thickness (MRT) and retinal vascularization (Allvin et al., 2014; Dyer et al., 2022; Fieß et al., 2021; Samarawickrama et al., 2009; Tariq et al., 2011; Wang et al., 2006).

Following MLP infants into adulthood is crucial for a comprehensive understanding of their development. Hence, this study aims to assess ocular fundus morphology in comparison with full‐term adolescents and in relation to general growth in adolescents born MLP.

## 2 MATERIALS AND METHODS

### 2.1 Study participants

This study is a part of a prospective population‐based cohort study of 247 participants (110 girls, 137 boys) born MLP in Gothenburg, the Region Västra Götaland, Sweden, between 2002 and 2004. Exclusion criteria were chromosomal abnormalities, severe chronic disease, asphyxia at birth and/or severe malformations.

The present study was performed between 2019 and 2021 at the Sahlgrenska University Hospital in Gothenburg. Out of the original 247 participants born MLP, one had passed away, another two had been excluded from the study based on having a neurological and a chromosomal disorder and 23 had moved out of the Region Västra Götaland. Invitations were consequently sent to 221 MLP adolescents. Seven declined participation and 164 did not respond to the invitation. The remaining 50 adolescents (26 girls, 24 boys) agreed to participate in the current study. A flowchart of the drop‐out rate is shown in Figure S1. The 50 participants had mean (standard deviation (SD)) GA, birth weight and birth length as follows: 35.0 (1.5) weeks, 2337 (655) gram and 45.1 (3.6) cm. The 171 non‐attending group showed the following means (SD): 35.2 (1.3) weeks, 2440 (462) gram and 46.1 (2.6) cm.

A control group born full term (GA ≥37 weeks) within the same years as the MLP group was recruited from primary schools, high schools and sports teams in Gothenburg.

### 2.2 Ethical approval

The study was conducted in accordance with the Declaration of Helsinki. Written consent was obtained from all participants after receiving oral and written information on the study. The study was approved by the Swedish Ethical Review Authority (#2019–04044).

### 2.3 Ophthalmological examinations

#### 2.3.1 Optical coherence tomography measurements

Optical coherence tomography (OCT) measurements were obtained under cycloplegia, with 0.85% cyclopentolate and 1.5% phenylephrine, using Topcon DRI OCT‐1 Triton Plus (Topcon Corporation, Tokyo, Japan). In both right eye (RE) and left eye (LE), three scans cantered on the macula and three scans cantered on the optic disc were obtained. The image with the best quality was selected. The following variables were obtained:

##### Macula

1. Macular RNFL, recorded in the nine Early Treatment of Diabetic Retinopathy Study (ETDRS) sectors A1–A9: central, inner superior, outer superior, inner inferior, outer inferior, inner temporal, outer temporal, inner nasal and outer nasal, expressed in μm. Additionally, the mean values of the inner and the outer sectors of the ETDRS chart were obtained.
2. MRT, recorded in the ETDRS sectors A1–A9 and presented as the mean values of the inner and outer sectors, as well as the central and total MRT, expressed in μm.
3. Macular retinal volume (MRV) expressed in mm^3^.

##### Optic disc

1. Peripapillary RNFL (ppRNFL) thickness, measured in four sectors (superior, inferior, temporal and nasal) and total thickness, expressed in μm.
2. Optic disc area, cup area, cup/disc (C/D) area ratio and rim area expressed in mm^2^.
3. Horizontal diameter of Bruch's membrane opening (BMO) expressed in mm.

#### 2.3.2 Grading of foveal hypoplasia

Foveal hypoplasia was graded, blinded, by two independent individuals (M.A.G. and A.L.), based on the fovea's different stages in its development in an arrested state. Grade 1 was defined as a shallow foveal pit, the presence of a wider outer nuclear layer (ONL) and an outer segment length elongation. Grade 2 displayed these characteristics but lacked a foveal pit. Grade 3 was defined as Grade 2 but without outer segment elongation. Grade 4 was defined as Grade 3 but without a wider ONL (Thomas et al., 2011).

#### 2.3.3 Refraction, visual acuity and total axial length

Refraction was measured under cycloplegia using an autorefractor (Topcon KR800, Topcon Medical Systems, Inc., Oakland, USA). For analysing OCT measurements, all participants with a refraction value of ≥6.00 diopter (D) spherical equivalent (SE) were excluded. Total axial length (TAL) was measured using Zeiss IOL Master 700, where a mean value of six measurements per eye was automatically given. Best‐corrected visual acuity (BCVA) was evaluated monocularly using the ETDRS chart at 4 m distance (Bailey & Lovie, 1976) and converted to logarithm of minimal angle of resolution (logMAR) values (Falkenstein et al., 2008).

### 2.4 Paediatric and growth variables

Data on GA, birth weight, birth length and birth HCF were retrieved from medical records at birth and converted to standard deviation scores (SDS) (Wikland et al., 2002). The number of individuals born small for gestational age (SGA), defined as birth weight and/or birth length ≤ −2 SDS (Karlberg & Albertsson‐Wikland, 1995), was noted. At 16 years of age, IGF‐I and IGFBP‐3 serum levels were taken in all participants and converted to SDS (Löfqvist et al., 2001). Additionally, height and HCF were obtained at 16 years of age in all participants.

### 2.5 Statistical analysis

For descriptive purposes, mean, SD, median and range were calculated. For comparison between groups, *t*‐test was used for continuous variables. The 95% confidence interval (CI) for the mean difference between the groups was based on the assumption of normality. The SD was based on Satterthwaite's approximation when variances were not equal (*p* < 0.05); otherwise, the SD was based on the pooled SD. A *p*‐value of <0.05 was considered statistically significant. However, for the macular RNFL thickness in the nine EDTRS sectors as well as the mean values of the inner and outer sectors, Bonferroni correction was used to account for multiple comparisons (*n* = 11) and a *p*‐value of <0.0045 was considered statistically significant. The difference between two groups was adjusted for TAL and sex using ANCOVA models, where mean difference between LS means with 95% CI and *p*‐value are presented in Tables S1 and S2.

Correlation analyses were made using Pearson partial correlations and Spearman rank partial correlations, depending on normality, adjusting for TAL and sex. In all participants, the GA, birth weight in SDS, birth length in SDS, birth HCF in SDS, height at assessment, HCF at assessment, IGF‐I levels in SDS at assessment, IGFBP‐3 levels in SDS at assessment, an IGF‐I/IGFBP‐3 ratio in SDS at assessment and BCVA at assessment were analysed to determine whether there was an association with the following ocular fundus morphology variables: central MRT, total ppRNFL thickness and C/D area ratio. Central MRT and total ppRNFL thickness were chosen for correlation analysis based on a significant difference found between the MLP group and controls, whereas the C/D area ratio was chosen for the optic disc parameters.

## 3 RESULTS

As described above, participants with a refraction ≥6.00 D SE were excluded for all OCT measurements. For three MLP adolescents, dilated refraction values were not obtained; consequently, non‐dilated values were used for exclusion. In total, three MLP individuals were excluded due to having refraction values as follows: +7.63 D SE in RE, +6.88 D SE in LE (dilated values), +10.88 D SE in RE, +11.13 D SE in LE (dilated values) and +5.75 D SE in RE, +6.50 D SE in LE (non‐dilated values), respectively.

As three out of 50 MLP individuals were excluded, 47 MLP individuals participated.

Demographic data, growth parameters and ocular background features of all participating adolescents, divided into the MLP group (*n* = 47) and the full‐term control group (*n* = 50), are shown in Table 1. The refraction of the 45 MLP participants with dilated measurements ranged from −3.13 to +5.00 D SE in RE and from −3.50 to +4.88 D SE in LE. The two MLP participants with non‐dilated measurements had refraction values of −0.38 D SE in RE, −0.75 D SE in LE and +0.25 D SE in RE, +2.88 D SE in LE, respectively. The TAL, which was obtained from 46 out of the 47 MLP participants, ranged from 21.3 to 25.4 mm RE and 20.6 to 25.5 mm in LE. The BCVA ranged from −0.30 to 0.16 logMAR in RE and −0.26 to 0.58 logMAR in LE.

**TABLE 1:** Demographic data, growth parameters and ocular background features among participants born moderate‐to‐late preterm (MLP) and controls born full‐term.

Variables	MLP group	Controls
Girls	24 (51%)	30 (40%)
*n* (%)	*n* = 47	*n* = 50
Boys	23 (49%)	20 (60%)
*n* (%)	*n* = 47	*n* = 50
Gestational age mean (SD)	34.9 (1.5)	40.3 (1.3)
Weeks	*n* = 47	*n* = 50
Age at assessment mean (SD)	16.5 (0.5)	16.7 (1.0)
Years	*n* = 47	*n* = 50
Birth weight mean (SD)	−1.05 (1.87)	−0.31 (0.89)
SDS	*n* = 47	*n* = 48
Birth length mean (SD)	−1.06 (2.17)	−0.43 (0.92)
SDS	*n* = 47	*n* = 47
Birth HCF mean (SD)	−1.87 (1.13)	−0.12 (0.73)
SDS	*n* = 46	*n* = 40
Height at assessment mean (SD)	171.8 (11.1)	171.5 (0.88)
cm	*n* = 47	*n* = 50
HCF at assessment mean (SD)	55.8 (2.24)	56.2 (1.63)
cm	*n* = 47	*n* = 50
Born SGA	15 (32%)	0 (0%)
*n* (%)	*n* = 47	*n* = 50
IGF‐I levels at assessment mean (SD)	0.44 (0.81)	0.70 (0.53)
SDS	*n* = 40	*n* = 41
IGFBP‐3 levels at assessment mean (SD)	0.36 (0.78)	0.93 (0.76)
SDS	*n* = 40	*n* = 41
BCVA right eye median (range)	−0.08 (−0.30 to 0.16)	−0.10 (−0.28 to 0.12)
logMAR	*n* = 47	*n* = 50
BCVA left eye median (range)	−0.08 (−0.26 to 0.58)	−0.10 (−0.30 to 0.2)
logMAR	*n* = 47	*n* = 50
Refraction^a^ right eye median (range)	+0.75 (−3.13 to +5.00)	+0.31(−3.75 to +2.38)
D SE	*n* = 47	*n* = 50
Refraction^a^ left eye median (range)	+0.75 (−3.50 to +4.88)	+0.50 (−4.00 to +2.25)
D SE	*n* = 47	*n* = 50
TAL right eye median (range)	23.2 (21.3 to 25.4)	23.7 (22.0 to 25.9)
mm	*n* = 46	*n* = 50
TAL left eye median (range)	23.1 (20.6 to 25.5)	23.6 (22.0 to 25.9)
mm	*n* = 46	*n* = 50

Abbreviations: BCVA, best‐corrected visual acuity; D, diopter; HCF, head circumference; IGFBP‐3, insulin growth factor‐binding protein‐3; IGF‐I, insulin growth factor‐I; logMAR, logarithm of minimal angle of resolution; NA, not applicable; SD, standard deviation; SDS, standard deviation score; SE, spherical equivalent; SGA, small for gestational age (birth weight and/or birth length ≤ −2 SDS); TAL, total axial length.

^a^ Under cycloplegia, *n* = 45; without cycloplegia, *n* = 2.

### 3.1 Optical coherence tomography measurements

#### 3.1.1 Optical coherence tomography of the macula

**FIGURE 1:**
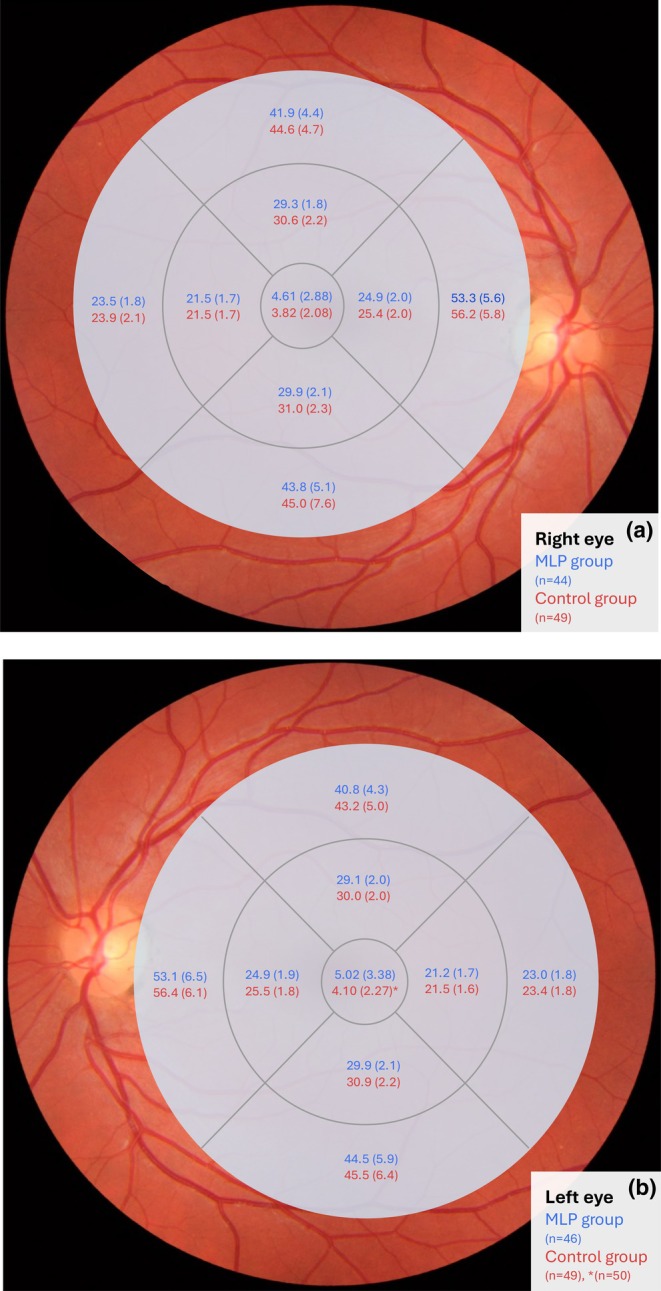
(a, b) Macular retinal nerve fibre layer (RNFL) thickness in the nine Early Treatment of Diabetic Retinopathy Study (EDTRS) areas of the right eye (a) and left eye (b), expressed in μm and presented as mean ± standard deviation, in the moderate‐to‐late preterm (MLP) group and the full‐term control group.

The MRT and MRV measurements are presented in Table 2. The number of participants excluded due to artefacts is found for each variable. The macular RNFL thickness in the nine EDTRS sectors is shown in Figure 1a,b, as well as in Tables S1 and S2. After adjusting for sex and TAL, as well as applying the Bonferroni correction, no significant difference was found between the groups regarding macular RNFL thickness.

**TABLE 2:** Macular retinal thickness and volume in adolescents born moderate‐to‐late preterm (MLP), compared with full‐term controls.

Variables	Eye	MLP group	Controls	*p*‐value	*p*‐value adjusted^a^	Difference between groups mean (95% CI)
MRT, total Mean (SD) μm	RE	286.5 (12.0) (*n* = 44)	286.8 (13.2) (*n* = 49)	0.90	0.16	−0.322 (−5.551; 4.907)
	LE	286.5 (11.7) (*n* = 46)	286.9 (13.5) (*n* = 49)	0.90	0.18	−0.334 (−5.504; 4.836)
MRT, central Mean (SD) μm	RE	249.7 (21.0) (*n* = 44)	239.9 (16.4) (*n* = 49)	**0.013**	**0.019**	9.81 (2.10; 17.52)
	LE	250.0 (21.6) (*n* = 46)	242.0 (19.2) (*n* = 50)	0.059	0.066	7.96 (−0.32; 16.24)
MRT, inner mean Mean (SD) μm	RE	318.9 (13.1) (*n* = 44)	317.8 (14.3) (*n* = 49)	0.70	0.63	1.10 (−4.57; 6.76)
	LE	318.9 (12.7) (*n* = 46)	318.1 (14.8) (*n* = 49)	0.77	0.58	0.831 (−4.798; 6.461)
MRT, outer mean Mean (SD) μm	RE	278.8 (12.8) (*n* = 44)	279.3 (13.9) (*n* = 49)	0.87	0.12	−0.450 (−5.987; 5.086)
	LE	278.2 (12.2) (*n* = 46)	279.3 (14.0) (*n* = 49)	0.69	0.088	−1.08 (−6.44; 4.28)
MRV Mean (SD) mm^3^	RE	8.10 (0.34) (*n* = 44)	8.11 (0.38) (*n* = 49)	0.91	0.16	−0.009 (−0.157; 0.139)
	LE	8.10 (0.33) (*n* = 46)	8.11 (0.38) (*n* = 49)	0.90	0.18	−0.009 (−0.155; 0.137)

*Note*: Significant *p*‐values are shown in bold values.

Abbreviations: CI, confidence interval; LE, left eye; MRT, macular retinal thickness; MRV, macular retinal volume; RE, right eye; SD, standard deviation.

^a^ The difference between the groups was adjusted for total axial length and sex. The adjusted means are shown in Tables S1 and S2.

#### 3.1.2 Optical coherence tomography of the optic disc

**FIGURE 2:**
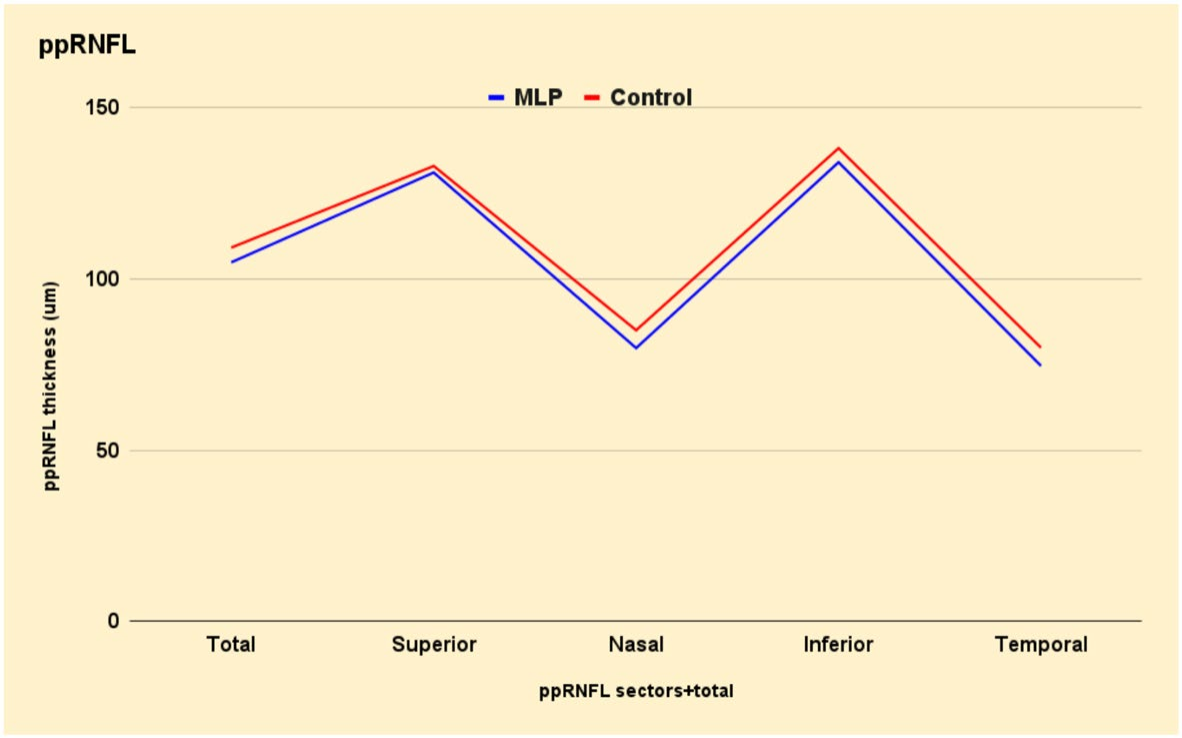
Average thickness of the peripapillary retinal nerve fibre layer (ppRNFL) in total and in the four sectors, expressed in μm and presented as mean ± standard deviation in the right eye, among adolescents born moderate‐to‐late preterm (MLP) and full‐term controls.

The optic disc area, cup area, C/D area ratio, rim area, horizontal diameter of BMO and ppRNFL thickness are shown in Table 3. To further present the ppRNFL, the thickness in total and in the four sectors in RE is shown in Figure 2.

**TABLE 3:** Optic disc parameters and peripapillary retinal nerve fibre layer (ppRNFL) thickness in adolescents born moderate‐to‐late preterm (MLP), compared with full‐term controls.

Variables	Eye	MLP group	Controls	*p*	*p* adjusted^a^	Difference between groups mean (95% CI)
Disc area Mean (SD) mm^2^	RE	1.92 (0.35) (*n* = 43)	1.88 (0.40) (*n* = 48)	0.62	0.87	0.039 (−0.118; 0.197)
	LE	1.91 (0.33) (*n* = 43)	1.93 (0.43) (*n* = 45)	0.85	0.43	−0.015 (−0.179; 0.148)
Cup area Mean (SD) mm^2^	RE	0.423 (0.335) (*n* = 43)	0.470 (0.425) (*n* = 48)	0.56	0.70	−0.047 (−0.208; 0.113)
	LE	0.446 (0.362) (*n* = 43)	0.450 (0.405) (*n* = 45)	0.96	0.95	−0.004 (−0.167; 0.159)
Rim area Mean (SD) mm^2^	RE LE	1.50 (0.45) (*n* = 43)	1.42 (0.34) (*n* = 48)	0.29	0.59	0.089 (−0.077; 0.255)
		1.47 (0.43) (*n* = 43)	1.48 (0.43) (*n* = 45)	0.88	0.50	−0.014 (−0.196; 0.168)
C/D area ratio Mean (SD)	RE LE	0.221 (0.159) (*n* = 43)	0.233 (0.180) (*n* = 48)	0.74	0.99	−0.012 (−0.083; 0.059)
		0.230 (0.172) (*n* = 43)	0.224 (0.164) (*n* = 45)	0.88	0.71	0.006 (−0.066; 0.077)
Horizontal diameter of BMO Mean (SD) mm	RE LE	1.52 (0.18) (*n* = 45)	1.50 (0.17) (*n* = 50)	0.47	0.73	0.025 (−0.045; 0.096)
		1.51 (0.16) (*n* = 46)	1.53 (0.16) (*n* = 47)	0.59	0.23	−0.018 (−0.084; 0.048)
ppRNFL, total Mean (SD) μm	RE LE	104.3 (8.5) (*n* = 38)	109.1 (8.3) (*n* = 40)	**0.015**	**0.0011**	−4.76 (−8.55; −0.97)
		104.6 (9.5) (*n* = 40)	106.8 (8.5) (*n* = 38)	0.28	0.051	−2.22 (−6.28; 1.85)
ppRNFL, superior Mean (SD) μm	RE LE	130.3 (12.7) (*n* = 38)	132.9 (13.8) (*n* = 40)	0.38	0.35	−2.64 (−8.64; 3.36)
		132.7 (13.0) (*n* = 40)	135.6 (12.1) (*n* = 38)	0.30	0.15	−2.96 (−8.62; 2.71)
ppRNFL, nasal Mean (SD) μm	RE LE	79.4 (13.2) (*n* = 38)	85.0 (10.8) (*n* = 40)	**0.043**	**0.0012**	−5.63 (−11.07; −0.19)
		77.0 (13.8) (*n* = 40)	81.7 (13.4) (*n* = 38)	0.13	**0.025**	−4.76 (−10.89; 1.37)
ppRNFL, inferior Mean (SD) μm	RE LE	133.5 (14.4) (*n* = 38)	138.1 (12.8) (*n* = 40)	0.14	**0.0031**	−4.55 (−10.70; 1.60)
		136.2 (18.5) (*n* = 40)	135.5 (13.8) (*n* = 38)	0.85	0.29	0.725 (−6.656; 8.106)
ppRNFL, temporal Mean (SD) μm	RE	74.4 (10.2) (*n* = 38)	79.9 (11.2) (*n* = 40)	**0.028**	0.054	−5.48 (−10.35; −0.60)
	LE	72.3 (10.4) (*n* = 40)	74.5 (9.9) (*n* = 38)	0.35	0.58	−2.17 (−6.77; 2.42)

*Note*: Significant *p*‐values are shown in bold values.

Abbreviations: BMO, Bruch's membrane opening; C/D, cup/disc; CI, confidence interval; LE, left eye; RE, right eye; SD, standard deviation.

^a^ The difference between the groups was adjusted for total axial length and sex. The adjusted means are shown in Tables S1 and S2.

#### 3.1.3 Grading of foveal hypoplasia

No difference was observed in foveal grading between the MLP group and the controls, with only Grade 1 foveal hypoplasia identified. In the MLP group (*n* = 45 in RE, *n* = 47 in LE), Grade 1 foveal hypoplasia was found in one case in both eyes, in two cases in LE and in three cases in RE. In the control group (*n* = 50), Grade 1 foveal hypoplasia was observed in two cases in both eyes and in two in LE.

### 3.2 Correlations

A weak association between total ppRNFL thickness and IGF‐I levels was found in all participants (*r* = 0.28, *p* = 0.032, in RE; *r* = 0.27, *p* = 0.048, in LE; Pearson's correlation analysis). Moreover, the C/D area ratio at assessment was related to birth weight (*r* = −0.44, *p* = 0.0006, in RE; *r* = −0.30, *p* = 0.026, in LE). In addition, there was a weak association between the IGF‐I/IGFBP‐3 ratio and central MRT in RE (*r* = 0.30, *p* = 0.022) but not in LE. No correlations were noted between the ocular fundus morphology variables and GA, birth length, birth HCF, height at assessment, HCF at assessment or BCVA.

## 4 DISCUSSION

This prospective and population‐based study suggests that general growth and being born MLP may impact ocular fundus morphology in adolescence. The study found MLP adolescents to have thinner nasal and total ppRNFL thickness, as well as greater central MRT, in comparison with full‐term controls. Furthermore, growth factor levels were shown to be associated with total ppRNFL thickness and central MRT. In addition, the present study found a correlation between birth weight and optic disc parameters in adolescence.

As mentioned previously, preterm birth may halt macular development, leading to migration disturbances (Hendrickson & Yuodelis, 1984). Lower GA has been shown to correlate with increased central MRT and foveal minimum thickness. Accordingly, thicker central MRT with a smaller foveal pit is well known in extremely and very preterm children and adolescents, with or without the presence of ROP (Åkerblom et al., 2011; Balasubramanian et al., 2019; Bowl et al., 2019; Fieß et al., 2022; Jørgensen et al., 2024; Park & Oh, 2012; Pétursdóttir et al., 2024; Tariq et al., 2011; Torres‐Peña et al., 2022; Venkataraman et al., 2023). Furthermore, these structural retinal anomalies have been found to be related to decreased visual function (Balasubramanian et al., 2019; Bowl et al., 2019). In the present study, no correlation was found between OCT measurements and BCVA at 16 years of age, aligning with the findings of Jørgensen et al. (2024) in their research on adults born preterm with very low birth weight.

The current study has found that MLP adolescents, with no history of ROP, may have thicker central MRT compared with full‐term controls at 16 years of age. This was already seen at 8 years of age in our previous study including MLP children recruited from the same cohort as in the current study (Raffa et al., 2016). Furthermore, a study performed in Saudi Arabia showed greater central MRT among MLP children aged 5–10 years (Raffa et al., 2021). Our previous studies have also shown greater foveal minimum thickness at 12 years of age (Raffa et al., 2017) and increased central foveal thickness at 16 years of age in MLP individuals compared with full‐term controls (Ovik et al., 2024). Regarding total MRT, inner and outer mean MRT, and MRV, there was no significant difference between the MLP group and controls in the present study. Similarly, concerning MRV, a previous study of individuals born extremely preterm found no difference compared with full‐term controls at 5–16 years of age (Åkerblom et al., 2011).

A previous study has shown thinner macular RNFL thickness in children born at GA <32 weeks in comparison with full‐term controls (Wang et al., 2012). By contrast, the present study of MLP adolescents found no difference between the MLP and control group after adjusting for sex and TAL, as well as for multiple comparisons.

Thinner ppRNFL thickness has been found in children and adults born extremely and very preterm (Kulmala et al., 2025; Pétursdóttir et al., 2024; Rothman et al., 2015; Wang et al., 2012). In the present study, the MLP group exhibited thinner ppRNFL thickness, and in particular thinner nasal ppRNFL thickness, in comparison with the full‐term group. Notably, thinning of the nasal and inferior sectors of the ppRNFL was also reported by Kulmala et al. (2025) in their study of adults born preterm with a birth weight under 1500 gram. There was already a trend towards a thinner ppRNFL in our MLP cohort at 12 years of age (Raffa et al., 2017). Thinner ppRNFL thickness has been associated with brain lesions, as well as with reduced cognitive and motor skills (Rothman et al., 2015). Therefore, the observed difference in ppRNFL thickness between the MLP group and controls warrants further consideration.

As previously mentioned, levels of IGF‐I have been shown to impact ocular development (Allvin et al., 2014; Hellström et al., 2003; Lind et al., 2018; Zhang et al., 2018). In our previous OCT study on MLP children at 8 years of age, a negative association between central retinal thickness and the change in IGF‐I levels from birth to 8 years of age was found. In similarity, in the present study at 16 years of age, central MRT showed a weak association with the IGF‐I/IGFBP‐3 levels ratio.

In our MLP study at 8 years of age, lower IGF‐I levels were found to be correlated with larger optic cup areas (Raffa et al., 2016). Furthermore, in the present study at 16 years of age, there was a weak association between lower IGF‐I levels and thinner total ppRNFL thickness. Previous studies have also found ppRNFL thickness to be associated with other growth parameters including birth weight, birth HCF (Wang et al., 2006) and fetal HCF (Dyer et al., 2022). In the present study, ppRNFL thickness did not correlate with weight, length or HCF at birth.

In the present study, greater C/D ratio was associated with lower birth weight. This is in accordance with a previous study at 12 years of age, where children born with low birth weight showed decreased optic disc and cup diameter, as well as elevated C/D ratio (Samarawickrama et al., 2009). Furthermore, in our MLP study at 8 years of age, optic disc area correlated with birth length, weight at assessment and height at assessment. Also, the C/D area ratio was associated with birth HCF (Raffa et al., 2016). These findings suggest that general growth, including low birth weight, may affect the development of the optic disc from birth through adolescence.

A study of preterm‐born participants aged 18–52 years revealed a correlation between lower GA and increased incidence of foveal hypoplasia. Interestingly, MLP individuals were also found with foveal hypoplasia (Fieß et al., 2022). By contrast, no difference in foveal grading was found between the MLP group and controls at 16 years of age in the current study.

Limitations of the present study include the relatively small sample size due to the drop‐out rate from the original neonatal study. However, the present findings contribute valuable insights into retinal morphological features in a group of individuals in whom ocular development is rarely studied. Additionally, as being a prospective and population‐based cohort study including a full‐term control group in the same age range, the study enhances the validity of its findings.

In conclusion, MLP birth may be associated with changes in ocular fundus morphology in adolescence, including thicker central MRT and thinner ppRNFL thickness. Consequently, MLP individuals were shown to have similar retinal findings as previously reported in extremely and very preterm individuals, underscoring the importance of additional studies in this group. Furthermore, the present study proposes that general growth may impact ocular fundus morphology. The ppRNFL thickness and the central MRT were shown to have a weak association with growth factor levels. Additionally, the study suggests that low birth weight may affect optic disc parameters. Larger population‐based studies are of great interest to confirm these findings and elucidate the role of general growth in ocular development, aiding clinical management in the MLP population.

## AUTHOR CONTRIBUTIONS

All authors were responsible for the concept and design of the study, data interpretation and manuscript drafting and revision, and have approved the final manuscript as submitted.

## FUNDING INFORMATION

M.A.G., E.A., A.L. and J.D. were supported by grants from Gothenburg Medical Society, the W. & M. Lundgren Vetenskapsfond II, the Swedish Research Society, the Foundation De Blindas Vänner and the Swedish State under the ALF agreement between the Swedish government and the country councils (grants No. ALFGBG‐11626, ALFGBG‐11869, ALFGBG‐211671, ALFGBG‐445021, ALFGBG 509761, ALFGBG 672501, ALFGBG 71933, ALGBG‐719711, ALFGBG‐772951, ALFGBG‐932558 and ALFGBG‐965041).

## CONFLICT OF INTEREST STATEMENT

The authors have no conflicts of interest relevant to this article to disclose.

## DISCLOSURE

M.A.G. has received lecture fees from Bayer, and J.D. has received lecture fees from Pfizer, Nestlé, Merck and Novo Nordisk. The remaining authors have no financial disclosures.

## Supporting information


**Figure S1.** A flowchart presenting the drop‐out rate from the original moderate‐to‐late preterm (MLP) population‐based cohort consisting of 247 participants (110 girls, 137 boys) born in Gothenburg, Sweden, between 2002 and 2004. Exclusion criteria were chromosomal abnormalities, severe chronic disease, asphyxia at birth and/or severe malformations. For analysing optical coherence tomography measurements, all participants with a refractive error of ≥ 6.00 diopter (D) spherical equivalent (SE) were excluded.

**Table S1.** Optical coherence tomography variables in adolescents born moderate‐to‐late preterm (MLP) and full‐term controls in right eye.

**Table S2.** Optical coherence tomography variables in adolescents born moderate‐to‐late preterm (MLP) and full‐term controls in left eye.
