# Supplementary figures and images for: Exploring ocular fundus morphology in relation to growth in adolescents born moderate‐to‐late preterm
Source: Acta Ophthalmol. 2025 Oct 3;104(3):e346–55. doi: 10.1111/aos.70011 (PMC13058674; doi:10.1111/aos.70011)

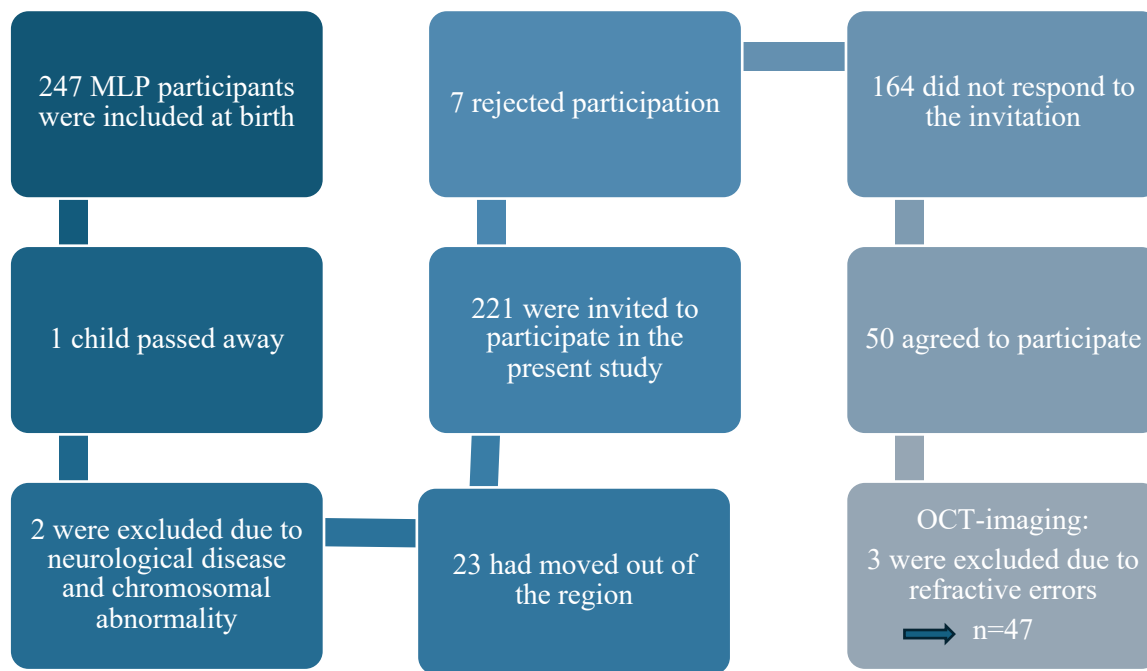

Supplement: Supplementary file 1 — Figure S1. A flowchart presenting the drop‐out rate from the original moderate‐to‐late preterm (MLP) population‐based cohort consisting of 247 participants (110 girls, 137 boys) born in Gothenburg, Sweden, between 2002 and 2004. Exclusion criteria were chromosomal abnormalities, severe chronic disease, asphyxia at birth and/or severe malformations. For analysing optical coherence tomography measurements, all participants with a refractive error of ≥ 6.00 diopter (D) spherical equivalent (SE) were excluded. [file AOS-104-e346-s002.pdf]
