# Supplementary material for: Exploring ocular fundus morphology in relation to growth in adolescents born moderate‐to‐late preterm
Source: Acta Ophthalmol. 2025 Oct 3;104(3):e346–55. doi: 10.1111/aos.70011 (PMC13058674; doi:10.1111/aos.70011)
Supplement: Supplementary file 2 — Table S1. Optical coherence tomography variables in adolescents born moderate‐to‐late preterm (MLP) and full‐term controls in right eye. [file AOS-104-e346-s001.docx]

**Supplementary Table 1.** Optical coherence tomography variables in adolescents born moderate-to-late preterm (MLP) and full-term controls in right eye.

| **Variable**  **right eye** | **MLP group**  **mean (SD)**  **median (range)** | **MLP group**  **adjusted means^1^ SEM (95% CI)** | **Controls**  **mean (SD)**  **median (range)** | **Controls**  **adjusted means^1^ SEM (95% CI)** | **p-value** | **p-value**  **adjusted^1^** | **Difference between groups**  **adjusted means (95% CI)** |
| --- | --- | --- | --- | --- | --- | --- | --- |
| **Disc area** | 1.92 (0.35)  1.95 (1.18; 2.91) n=43 | 1.91  0.06 (1.79-2.03) | 1.88 (0.40)  1.84 (1.42; 3.44) n=48 | 1.90  0.06 (1.78-2.01) | 0.62 | 0.87 | 0.014 (-0.154; 0.182) |
| **Cup area** | 0.423 (0.335)  0.39 (0; 1.26)  n=43 | 0.431  0.060 (0.312-0.550) | 0.470 (0.425)  0.325 (0; 1.88)  n=48 | 0.464  0.056 (0.353-0.575) | 0.56 | 0.70 | -0.033 (-0.200; 0.134) |
| **Rim area** | 1.50 (0.45)  1.39 (0.83; 2.91) n=43 | 1.48  0.06 (1.36-1.61) | 1.42 (0.34)  1.34 (0.53; 2.62) n=48 | 1.43  0.06 (1.32-1.55) | 0.29 | 0.59 | 0.048 (-0.128; 0.224) |
| **C/D area ratio** | 0.221 (0.159)  0.21 (0; 0.6)  n=43 | 0.227  0.027 (0.174-0.280) | 0.233 (0.180)  0.2 (0; 0.72)  n=48 | 0.228  0.025 (0.178-0.277) | 0.74 | 0.99 | -0.000 (-0.075; 0.074) |
| **Horizontal BMO** | 1.52 (0.18)  1.54 (1.08; 2.07) n=45 | 1.51  0.03 (1.46-1.57) | 1.50 (0.17)  1.5 (1.19; 2)  n=50 | 1.50  0.02 (1.45-1.55) | 0.47 | 0.73 | 0.013 (-0.061; 0.087) |
| **ppRNFL,**  **total** | 104.3 (8.5)  104 (87; 124)  n=38 | 103.4  1.4 (100.7-106.1) | 109.1 (8.3)  109 (88; 128)  n=40 | 110.0  1.3 (107.4-112.6) | **0.015** | **0.0011** | -6.59 (-10.47; -2.71) |
| **ppRNFL,**  **superior** | 130.3 (12.7)  132 (94; 162)  n=38 | 130.0  2.3 (125.4-134.5) | 132.9 (13.8)  133.5 (105; 160) n=40 | 133.0  2.2 (128.6-137.4) | 0.38 | 0.35 | -3.05 (-9.53; 3.44) |
| **ppRNFL,**  **nasal** | 79.4 (13.2)  79 (58; 120)  n=38 | 77.6  1.9 (73.9-81.4) | 85.0 (10.8)  87 (63; 106)  n=40 | 86.7  1.8 (83.1-90.3) | **0.043** | **0.0012** | -9.04 (-14.37; -3.70) |
| **ppRNFL,**  **inferior** | 133.5 (14.4)  129 (111; 173)  n=38 | 131.2  2.1 (127.1-135.3) | 138.1 (12.8)  137.5 (102; 162) n=40 | 140.2  2.0 (136.2-144.1) | 0.14 | **0.0031** | -8.94 (-14.77; -3.12) |
| **ppRNFL,**  **temporal** | 74.4 (10.2)  72 (59; 107)  n=38 | 74.8  1.8 (71.1-78.4) | 79.9 (11.2)  79 (60; 110)  n=39 | 79.9  1.8 (76.3-83.4) | **0.028** | 0.054 | -5.10 (-10.27; 0.08) |
| **Macular RNFL, central** | 4.61 (2.88)  4 (0; 12)  n=44 | 4.71  0.38 (3.95-5.46) | 3.82 (2.08)  3 (1; 10)  n=49 | 3.71  0.35 (3.00-4.41) | 0.13^2^ | 0.064^2^ | 0.997 (-0.060; 2.055) |
| **Macular RNFL, inner superior** | 29.3 (1.8)  29 (26; 33)   n=44 | 29.6  0.3 (29.0-30.2) | 30.6 (2.2) 3  1 (26; 34)  n=49 | 30.3  0.3 (29.8-30.9) | **0.0037**^2^ | 0.089^2^ | -0.719 (-1.552; 0.113) |
| **Macular RNFL, outer superior** | 41.9 (4.4)  41 (34; 56)  n=44 | 42.1  0.7 (40.7-43.5) | 44.6 (4.7)  44 (37; 55)  n=49 | 44.5  0.6 (43.2-45.8) | 0.0048^2^ | 0.014^2^ | -2.44 (-4.38; -0.51) |
| **Macular RNFL, inner nasal** | 24.9 (2.0)  25 (21; 30)  n=44 | 25.2  0.3 (24.6-25.7) | 25.4 (2.0)  26 (20; 29)  n=49 | 25.2  0.3 (24.6-25.7) | 0.27^2^ | 0.97^2^ | -0.015 (-0.819; 0.789) |
| **Macular RNFL, outer nasal** | 53.3 (5.6)  53 (41; 66)  n=44 | 54.0  0.9 (52.3-55.7) | 56.2 (5.8)  55 (46; 71)  n=49 | 55.7  0.8 (54.1-57.3) | 0.014^2^ | 0.16^2^ | -1.72 (-4.13; 0.68) |
| **Macular RNFL, inner inferior** | 29.9 (2.1)  30 (26; 35)  n=44 | 30.1  0.3 (29.4-30.8) | 31.0 (2.3)  31 (26; 36)  n=49 | 30.8  0.3 (30.2-31.4) | 0.020^2^ | 0.15^2^ | -0.699 (-1.652; 0.255) |
| **Macular RNFL, outer inferior** | 43.8 (5.1)  44 (32; 56)  n=44 | 44.2  1.0 (42.2-46.3) | 45.0 (7.6)  44 (20; 75)  n=49 | 44.7  1.0 (42.8-46.6) | 0.37^2^ | 0.73^2^ | -0.500 (-3.371; 2.371) |
| **Macular RNFL, inner temporal** | 21.5 (1.7)  22 (17; 24)  n=44 | 21.6  0.3 (21.1-22.1) | 21.5 (1.7)  22 (18; 24)  n=49 | 21.5  0.2 (21.0-22.0) | 0.93^2^ | 0.76^2^ | 0.114 (-0.630; 0.857) |
| **Macular RNFL, outer temporal** | 23.5 (1.8)  24 (19; 26)  n=44 | 23.4  0.3 (22.8-23.9) | 23.9 (2.1)  24 (19; 27)  n=49 | 24.1  0.3 (23.6-24.7) | 0.27^2^ | 0.066^2^ | -0.759 (-1.569; 0.051) |
| **Macular RNFL, mean inner** | 26.4 (1.5)  26.3 (23.5; 30)  n=44 | 26.6  0.2 (26.1-27.1) | 27.1 (1.7)  27 (23; 30.5)  n=49 | 26.9  0.2 (26.5-27.4) | 0.037^2^ | 0.34^2^ | -0.322 (-0.990; 0.346) |
| **Macular RNFL, mean outer** | 40.2 (4.5)  40.8 (27.5; 50)  n=44 | 40.7  0.6 (39.4-42.0) | 42.6 (4.2)  42 (35.5; 56)  n=49 | 42.3  0.6 (41.2-43.5) | 0.011^2^ | 0.075^2^ | -1.63 (-3.43; 0.17) |
| **MRT,**  **central** | 249.7 (21.0)  251.5 (205; 303) n=44 | 249.7  3.0 (243.8-255.6) | 239.9 (16.4)  239 (209; 277)  n=49 | 239.8  2.8 (234.3-245.3) | **0.013** | **0.019** | 9.94 (1.69; 18.18) |
| **MRT,**  **inner** **mean** | 318.9 (13.1)  316.5 (296.8; 346.8)  n=44 | 317.6  2.1 (313.4-321.7) | 317.8 (14.3)  317.3 (290; 357) n=49 | 319.0  2.0 (315.1-322.9) | 0.70 | 0.63 | -1.43 (-7.25; 4.40) |
| **MRT,**  **outer** **mean** | 278.8 (12.8)  277.3 (254.5; 302.8) n=44 | 276.8  1.9 (273.0-280.6) | 279.3 (13.9)  277.8 (249; 325) n=49 | 281.1  1.8 (277.5-284.6) | 0.87 | 0.12 | -4.25 (-9.59; 1.08) |
| **MRT,**  **total** | 286.5 (12.0)  284.9 (264.6; 311.5) n=44 | 284.7  1.8 (281.0-288.3) | 286.8 (13.2)  285.6 (258.3; 328.8) n=49 | 288.4  1.7 (285.0-291.8) | 0.90 | 0.16 | -3.69 (-8.82; 1.45) |
| **MRV** | 8.10 (0.34)  8.06 (7.48; 8.81) n=44 | 8.05  0.05 (7.95-8.15) | 8.11 (0.38)  8.08 (7.3; 9.3)  n=49 | 8.15  0.05 (8.06-8.25) | 0.91 | 0.16 | -0.104 (-0.249; 0.041) |

Abbreviations: BMO = Bruch’s membrane opening; C/D = cup/disc; CI = confidence interval; MLP = moderate-to-late preterm; MRT = macular retinal thickness; MRV = macular retinal volume; ppRNFL = peripapillary retinal nerve fibre layer; RNFL = retinal nerve fibre layer; SD = standard deviation.

^1^The difference between the groups was adjusted for total axial length and sex.
^2^Bonferroni correction was used to account for multiple comparisons and a p-value of <0.0045 was considered statistically significant.
