# Supplementary material for: Exploring ocular fundus morphology in relation to growth in adolescents born moderate‐to‐late preterm
Source: Acta Ophthalmol. 2025 Oct 3;104(3):e346–55. doi: 10.1111/aos.70011 (PMC13058674; doi:10.1111/aos.70011)
Supplement: Supplementary file 3 — Table S2. Optical coherence tomography variables in adolescents born moderate‐to‐late preterm (MLP) and full‐term controls in left eye. [file AOS-104-e346-s003.docx]

**Supplementary Table 2.** Optical coherence tomography variables in adolescents born moderate-to-late preterm (MLP) and full-term controls in left eye.

| **Variable**  **left eye** | **MLP group**  **mean (SD)**  **median (range)** | **MLP group**  **adjusted means^1^ SEM (95% CI)** | **Controls**  **mean (SD)**  **median (range)** | **Controls**  **adjusted means^1^ SEM (95% CI)** | **p-value** | **p-value**  **adjusted^1^** | **Difference between groups**  **adjusted means (95% CI)** |
| --- | --- | --- | --- | --- | --- | --- | --- |
| **Disc area** | 1.91 (0.33)  1.94 (1.22; 2.96)  n=43 | 1.88  0.06 (1.76-2.00) | 1.93 (0.43) 1  .88 (1.25; 3.53)  n=45 | 1.95  0.06 (1.84-2.07) | 0.85 | 0.43 | -0.068 (-0.238; 0.103) |
| **Cup area** | 0.446 (0.362)  0.36 (0; 1.48)  n=43 | 0.448  0.060 (0.330-0.567) | 0.450 (0.405)  0.32 (0; 2.03)  n=45 | 0.454  0.058 (0.339-0.568) | 0.96 | 0.95 | -0.005 (-0.174; 0.164) |
| **Rim area** | 1.47 (0.43)  1.48 (0.7; 2.96)  n=43 | 1.43  0.07 (1.30-1.57) | 1.48 (0.43)  1.4 (0.86; 3.53)  n=45 | 1.50  0.07 (1.37-1.63) | 0.88 | 0.50 | -0.065 (-0.256; 0.126) |
| **C/D area ratio** | 0.230 (0.172)  0.21 (0; 0.68)  n=43 | 0.236  0.026 (0.184-0.288) | 0.224 (0.164)  0.2 (0; 0.59)  n=45 | 0.222  0.025 (0.172-0.273) | 0.88 | 0.71 | 0.014 (-0.061; 0.088) |
| **Horizontal BMO** | 1.51 (0.16)  1.52 (1.19; 1.92)  n=46 | 1.50  0.02 (1.45-1.55) | 1.53 (0.16)  1.51 (1.14; 2.01)  n=47 | 1.54  0.02 (1.49-1.59) | 0.59 | 0.23 | -0.041 (-0.108; 0.027) |
| **ppRNFL,**  **total** | 104.6 (9.5)  104.5 (82; 123)  n=40 | 103.8  1.4 (101.1-106.5) | 106.8 (8.5)  106.5 (86; 132)  n=38 | 107.7  1.4 (104.9-110.5) | 0.28 | 0.051 | -3.94 (-7.89; 0.00) |
| **ppRNFL,**  **superior** | 132.7 (13.0)  134.5 (96; 162)  n=40 | 132.0  2.0 (128.0-136.0) | 135.6 (12.1)  135 (109; 173)  n=38 | 136.3  2.1 (132.1-140.4) | 0.30 | 0.15 | -4.22 (-10.06; 1.62) |
| **ppRNFL,**  **nasal** | 77.0 (13.8)  78 (49; 115)  n=40 | 75.9  2.1 (71.7-80.1) | 81.7 (13.4)  81.5 (51; 110)  n=38 | 82.9  2.2 (78.6-87.2) | 0.13 | **0.025** | -7.00 (-13.09; -0.91) |
| **ppRNFL,**  **inferior** | 136.2 (18.5)  136.5 (77; 177)  n=40 | 134.1  2.3 (129.6-138.7) | 135.5 (13.8)  136 (103; 173)  n=38 | 137.7  2.4 (133.0-142.4) | 0.85 | 0.29 | -3.54 (-10.19; 3.11) |
| **ppRNFL,**  **temporal** | 72.3 (10.4)  70 (52; 100)  n=40 | 72.7  1.6 (69.4-76.0) | 74.5 (9.9)  72.5 (59; 100)  n=38 | 74.0  1.7 (70.7-77.4) | 0.35 | 0.58 | -1.33 (-6.09; 3.42) |
| **Macular RNFL, central** | 5.02 (3.38)  4.5 (0; 14)  n=46 | 5.12  0.42 (4.29-5.95) | 4.10 (2.27)  4 (1; 12)  n=50 | 3.95  0.39 (3.17-4.74) | 0.12^2^ | 0.051^2^ | 1.17 (-0.00; 2.34) |
| **Macular RNFL, inner superior** | 29.1 (2.0)  29 (25; 34)  n=46 | 29.5  0.3 (29.0-30.0) | 30.0 (2.0)  30 (25; 34)  n=49 | 29.7  0.3 (29.1-30.2) | 0.041^2^ | 0.67^2^ | -0.165 (-0.939; 0.609) |
| **Macular RNFL, outer superior** | 40.8 (4.3)  41 (31; 55)  n=46 | 41.3  0.7 (39.9-42.7) | 43.2 (5.0)  43 (31; 55)  n=49 | 42.9  0.7 (41.5-44.2) | 0.011^2^ | 0.11^2^ | -1.59 (-3.57; 0.39) |
| **Macular RNFL, inner nasal** | 24.9 (1.9)  25 (21; 29)  n=46 | 25.2  0.2 (24.7-25.7) | 25.5 (1.8)  26 (20; 29)  n=49 | 25.2  0.2 (24.8-25.7) | 0.11^2^ | 0.93^2^ | -0.032 (-0.702; 0.638) |
| **Macular RNFL, outer nasal** | 53.1 (6.5)  53 (38; 75)  n=46 | 54.1  0.9 (52.3-55.9) | 56.4 (6.1)  56 (47; 76)  n=49 | 55.6  0.9 (53.9-57.3) | 0.013^2^ | 0.25^2^ | -1.49 (-4.06; 1.07) |
| **Macular RNFL, inner inferior** | 29.9 (2.1)  30 (25; 35)  n=46 | 30.2  0.3 (29.5-30.8) | 30.9 (2.2)  31 (27; 37)  n=49 | 30.8  0.3 (30.1-31.4) | 0.025^2^ | 0.20^2^ | -0.586 (-1.493; 0.321) |
| **Macular RNFL, outer inferior** | 44.5 (5.9)  44.5 (32; 60)  n=46 | 45.1  0.9 (43.2-47.0) | 45.5 (6.4)  44 (34; 74)  n=49 | 45.2  0.9 (43.4-47.0) | 0.44^22^ | 0.97^2^ | -0.056 (-2.723; 2.611) |
| **Macular RNFL, inner temporal** | 21.2 (1.7)  22 (16; 24)  n=46 | 21.4  0.2 (20.9-21.9) | 21.5 (1.6)  22 (17; 24)  n=49 | 21.4  0.2 (20.9-21.9) | 0.42^2^ | 0.95^2^ | -0.024 (-0.726; 0.679) |
| **Macular RNFL, outer temporal** | 23.0 (1.8)  23 (18; 26)  n=46 | 23.0  0.3 (22.5-23.5) | 23.4 (1.8)  24 (18; 26)  n=49 | 23.4  0.2 (22.9-23.9) | 0.32^2^ | 0.27^2^ | -0.407 (-1.135; 0.321) |
| **Macular RNFL, mean inner** | 26.3 (1.5)  26.3 (23; 29.8)  n=46 | 26.6  0.2 (26.1-27.0) | 27.0 (1.5)  27 (22.8; 30.5)  n=49 | 26.8  0.2 (26.4-27.2) | 0.031^2^ | 0.50^2^ | -0.202 (-0.798; 0.394) |
| **Macular RNFL, mean outer** | 40.3 (4.0)  40.1 (31; 52.3)  n=46 | 40.9  0.6 (39.6-42.1) | 42.1 (4.3)  41 (35; 57.5)  n=49 | 41.8  0.6 (40.6-43.0) | 0.038^2^ | 0.31^2^ | -0.900 (-2.658; 0.858) |
| **MRT,**  **central** | 250.0 (21.6)  250 (213; 310)  n=46 | 250.0  3.1 (243.8-256.3) | 242.0 (19.2)  239.5 (197; 285)  n=50 | 241.8  3.0 (235.9-247.7) | 0.059 | 0.066 | 8.25 (-0.54; 17.05) |
| **MRT,**  **inner** **mean** | 318.9 (12.7)  318.8 (294.3; 349.3)  n=46 | 317.6  2.1 (313.5-321.8) | 318.1 (14.8)  317.8 (289.8; 362.8)  n=49 | 319.3  2.0 (315.3-323.2) | 0.77 | 0.58 | -1.66 (-7.56; 4.23) |
| **MRT,**  **outer** **mean** | 278.2 (12.2)  277.4 (252; 301.3)  n=46 | 276.3  1.9 (272.6-280.1) | 279.3 (14.0)  278.3 (248.5; 320.5)  n=49 | 281.0  1.8 (277.4-284.6) | 0.69 | 0.088 | -4.64 (-9.99; 0.71) |
| **MRV** | 286.5 (11.7)  284.8 (261.4; 311.6)  n=46 | 284.8  1.8 (281.1-288.5) | 286.9 (13.5)  285.1 (258; 325.3)  n=49 | 288.4  1.8 (284.9-291.9) | 0.90 | 0.18 | -3.57 (-8.80; 1.66) |

Abbreviations: BMO = Bruch’s membrane opening; C/D = cup/disc; CI = confidence interval; MLP = moderate-to-late preterm; MRT = macular retinal thickness; MRV = macular retinal volume; ppRNFL = peripapillary retinal nerve fibre layer; RNFL = retinal nerve fibre layer; SD = standard deviation.

^1^The difference between the groups was adjusted for total axial length and sex.
^2^Bonferroni correction was used to account for multiple comparisons and a p-value of <0.0045 was considered statistically significant.
